# A polysaccharide-peptide with mercury clearance activity from dried fruiting bodies of maitake mushroom *Grifola frondosa*

**DOI:** 10.1038/s41598-018-35945-9

**Published:** 2018-12-04

**Authors:** Weiwei Zhang, Xuezhen Jiang, Shuang Zhao, Xiaojie Zheng, Jin Lan, Hexiang Wang, Tzi Bun Ng

**Affiliations:** 1 Institute of Medicinal Plant Development, Chinese Academy of Medical Sciences & Peking Union Medical College, Beijing, 100193 China; 2 State Key Laboratory for Agrobiotechnology and Department of Microbiology, China Agricultural University, Beijing, 100193 China; 3 Institute of Plant and Environment Protection, Beijing Academy of Agriculture and Forestry Sciences, Beijing, 100097 China; 4 School of Biomedical Sciences, Faculty of Medicine, The Chinese University of Hong Kong, Shatin, New Territories, Hong Kong China

## Abstract

Mercury is considered to be “a global pollutant” and raises concern worldwide. Once mercury enters the body, it will be distributed all over the body but will accumulate in the brain, kidney and liver. To date, no substance originating from edible fungi capable of adsorbing mercury has been reported. We found that the mushroom *Grifola frondosa* exhibited mercury adsorption capacity. A polysaccharide-peptide (GFPP), displaying the unique N-terminal amino acid sequence of APPGMHQKQQ and 7 partial sequences with high reliability obtained by LC-MS/MS, was isolated by hot-water extraction of its fruiting bodies followed by ion exchange chromatography and gel filtration chromatography. Two rat models were employed to determine the dose and the duration of HgCl_2_ treatment (given by acute administration or continuous treatment) to test if *G. frondosa* could promote mercury elimination. For rats subjected to acute treatment with HgCl_2_, both GFPP and *G. frondosa* fruiting bodies (GFFF) could accelerate the decline of blood mercury level, which fell precipitously by 50% on the second day. GFPP and GFFF also promoted elimination of the burden of mercury in the liver and kidneys. For rats receiving continuous HgCl_2_ treatment, *G. frondosa* prevented the progressive increase of blood mercury level, and kept the blood mercury level within a relatively stable range.

## Introduction

Mercury is an element in the natural environment. It existed in natural water, atmosphere and soil at very low concentrations before the rapid development of industries. Due to its wide industrial applications, a large amount of mercury was discharged into the air, water and soil as a pollutant which threatens health. Mercury with different valences can be present in solid, liquid and gas forms in the environment, and mercury in different states can enter the body through different channels, especially via respiration and dietary intake. In organisms, mercury exists in two forms, inorganic and organic^1^. Mercury mainly exists in the inorganic form in plants but in the organic form in animals and microbes.

According to the clinical manifestations, mercury could bring about various health hazards including neurotoxicity, reproductive toxicity and injury to the renal, hepatic, respiratory and immune systems^2^. Due to the high lipid solubility, organic mercury can traverse the blood brain barrier and accumulates in the brain^3,4^. Even when exposed to low levels of mercury the nervous system can be damaged. The symptoms of neurotoxicity include not only mental, language, and behavioral disorders, but also sensory abnormalities, mental retardation and ataxia. Organic mercury can also damage the fetus after traversing the placental barrier and affect the reproductive function after passing through the blood-testis barrier^5^. The reproductive effects of mercury on male animals are produced by affecting the sex gland and endocrine function. The nucleic acid and subcellular structure of the reproductive cells treated by mercury are adversely affected to different extents, leading to cytotoxicity, interfering with supply to the cells, reduced sperm count and decreased sperm motility^6^. The reproductive effects of mercury on female animals are mainly observed in an extended estrus cycle, resulting in an ovulation barrier and delayed corpus luteum formation^7,8^. Exposure to mercury can disrupt DNA synthesis in reproductive cells, retard cell division, alter subcellular structure, induce abnormal energy metabolism, destroy fertilizing ability, and undermine reproductive ability. The liver and kidney are the organs in which mercury accumulates resulting in acute injury and oxidative stress^9^.

Mercury poisoning has been studied in humans and a diversity of animals encompassing monkeys, pigs, sheep, cows, horses, dogs, cats, rabbits, mice, chickens, ducks, quails, fish, and other animals. Among these animals, rats were the main subjects. There are also numerous studies on mercury removal from the body, in which two main categories of mercury drugs were used, chemicals and natural extracts. Chemical drugs against mercury include antioxidants like buthionine sulfoximing (BSO), glutathione (GSH), vitamin C, taurine and metal chelating agents such as 2, 3-dimercapto-1-propanol (BAL), 2, 3-dimercaptosuccinic acid (DMSA) and sodium 2, 3-dimercato-1-propaneulfonate (DMPS)^10–12^. DMSA and DMPS are good clinical drugs for removing organic and inorganic mercury, respectively. The side effects of chemical drugs have been proven. However, considering the toxic and side effects of chemical drugs, natural extracts for mercury removal have attracted considerable interest. Lycopene, procyandin and sulforaphane are antioxidants which protect against the oxidative damage and renal injury caused by mercury^13–15^. Luteolin, widespread in plants, not only curtailed the accumulation of renal mercury and promoted mercury excretion but also attenuated oxidative stress, inflammation and apoptosis^16–18^. Besides, *Smilax glabra*, schisandrin b and *Hippophae rhamnoides* oil were also reported to have a mercury-removing effect.

*Grifola frondosa*, a wild mushroom known worldwide, with a nice external appearance a delicious taste, and an abundance of nutrients, has a high value for food and medicinal purposes. The major ingredients of *G. frondosa* are polysaccharides. As an effective immune regulator, *G. frondosa* polysaccharide activates immune cells such as macrophages, NK cells and T lymphocytes, and also promotes secretion of cytokines including IL-2, IL-8, IL-12, TNF-α and others^19–21^. These immunoregulatory effects enable *G. frondosa* polysaccharide to have antitumor and antivirus capabilities. The hypoglycemic^22,23^, hypolipidemic^24^, hypotensive^25,26^, antioxidant^27^, hepatoprotective^28^, anti-radiation^29^, and antibacterial effects^30^ of *G. frondosa* polysaccharide have also been reported.

In this study, a polysaccharide-peptide was isolated from fruiting bodies of *G. frondosa* which was proved to have a mercury eliminating effect both *in vitro* and *in vivo*. This suggests that this edible mushroom could serve as a new resource for developing antidotes for heavy metal intoxication which can be used with safety on a long term basis.

## Results

### Purification of polysaccharide-peptide from *G. frondosa* fruiting bodies

**Figure 1:**
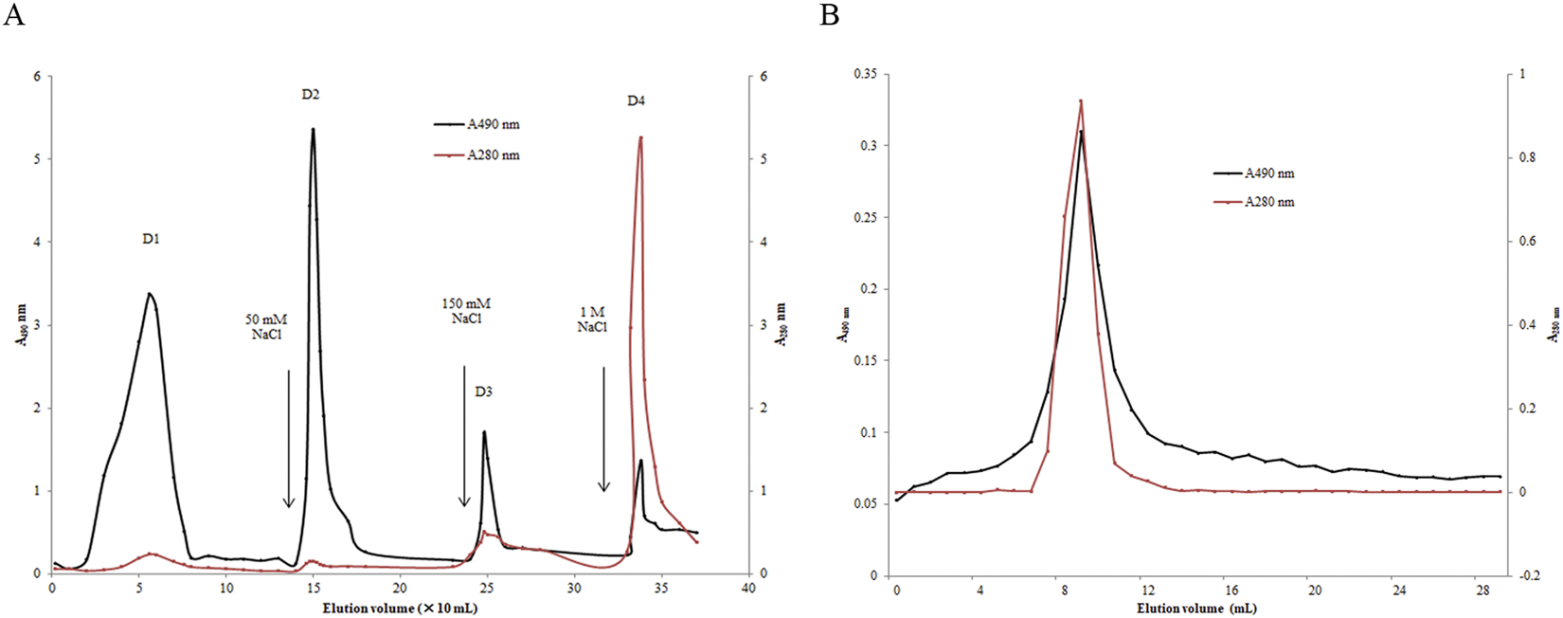
Elution profile of GFPP from (**A**) DEAE-cellulose ion exchange column and (**B**) gel filtration chromatograph. The protein content is displayed by A 280 nm in black. The polysaccharide content is displayed in red by A490 nm after phenol sulfuric acid reaction.

Through extraction with hot water twice, ethanol precipitation and drying, a crude product with mercury-removing capacity was obtained from *G. frondosa*. This crude product appeared to be a black powder with a yield of 9.1% and was designated as GFAS. GFAS was dissolved thoroughly and loaded on a DEAE- cellulose column. According to the elution profile (Fig. 1A), four fractions were eluted and fractions D1–D3 had a higher polysaccharide content but a lower protein content than fraction D4. All four fractions were collected and their mercury-removing effects *in vitro* were determined. The results showed that they all had mercury-removing activity. Fractions D1, D2, D3 and D4 possessed mercury clearance rates of 47.1%, 30.4%, 80.2% and 82.6%, respectively. Fraction D4, which exhibited the best activity, was then subjected to gel-filtration on Superdex 75. A single peak with a molecular weight of nearly 96 kDa was eluted (Fig. 1B). The single peak contained both polysaccharide and protein, with a high polysaccharide: protein ratio of 8.57:1. It was named *G. frondosa* polysaccharide peptide (GFPP). The mercury clearance rates of purified GFPP at the concentrations of 4 mg/mL, 2 mg/mL, 1 mg/mL, 0.5 mg/mL and 0.25 mg/mL were 74.2%, 56.6%, 31.7%, 12.6%, and 1.1% respectively. The polysaccharide contents and mercury clearance ratios of fractions from each chromatographic step were also evaluated and are summarized in Table 1.

**Table 1:** polysaccharide content and mercury clearance ratio.

Fraction	Yield (%)	Polysaccharide content (%)	Mercury clearance ratio (%)
GFAS	9.1	89.5	56.6 ± 3.4
GFAS-D4	1.6	87.5	82.6 ± 3.1
GFPP	0.9	89.6	88.2 ± 3.1

Results are represented as mean ± SD (n = 3).

### SDS-PAGE of GFPP and amino acid analysis

**Figure 2:**
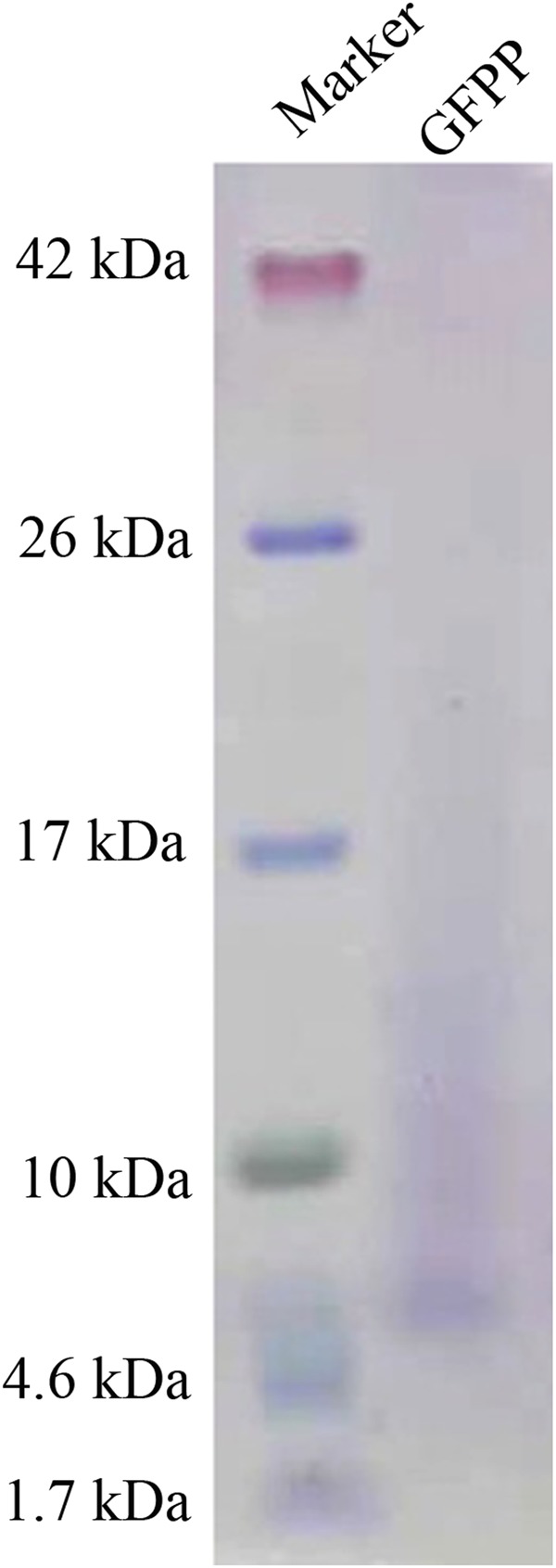
SDS-PAGE results on a 16% tricine gel. left lane: molecular mass standards. Right lane: peak fraction collected from the Superdex 75 which represents purified GFPP.

The purified GFPP was subjected to SDS-PAGE on a 16% tricine gel. The results displayed in Fig. 2 reveal that GFPP possessed a molecular mass of 6 kDa. Taken together with the molecular mass revealed by molecular sieve chromatography, GFPP appeared to be multimeric with 12 subunits. The trailing observed in electrophoresis also demonstrated that the component was a polysaccharide-peptide complex.

EDMAN sequencing of the GFPP band yielded the N- terminal sequence APPGMHQKQQ. The ESI-MS/MS of GFPP revealed seven peptides with higher reliability as follows: LVSLSCDPNHTFSIDGHSLTVIEADSVNLKPHTVDSIQIFAAQR, SLYDVDDDSTVITLADWYHLAAR, QAILVNDVFPSPLITGNKGDR, VGPAIPTADATLINGLGR, YSFVLNADQDVDNYWIR, SINTLNADLAVITVTK, and NFDGGVNSAILR.

### Affinity of GFPP toward different metal ions

Among all the metal ions detected, GFPP exhibited higher affinity toward Hg ions than other ions (Table 2). No affinity was detected with Ca^2+^, Cu^2+^, Al^3+^, and Fe^2+^ ions. Only slight affinity toward Mn^2+^, Mg^2+^ and Cd^2+^ ions was observed. GFPP exhibited specific adsorption of mercury. DMPS is a chelating agent which is often administered in acute or chronic intoxications by several heavy metals (e.g. cadmium, cobalt, lead and mercury), but it could also act as a depleter of physiologically important elements (e.g. potassium, magnesium, calcium)^31^.

**Table 2:** Affinities of GFPP toward different metal ions *in vivo*. Results are represented as mean ± SD (n = 3).

Metal ions	Mn^2+^	Ca^2+^	Mg^2+^	Cu^2+^	Cr^2+^	Al^3+^	Fe^2+^	Pb^2+^	Cd^2+^	Hg^2+^
Absorption ratio (%)	2.51 ± 0.40	−0.05 ± 0.01	16.45 ± 2.72	−1.76 ± 0.51	2.97 ± 0.22	−1.67 ± 0.13	0.50 ± 0.03	−7.16 ± 0.15	2.03 ± 0.18	34.32 ± 3.30

### Detoxifying effect of GFPP and GFFF on acute mercury poisoning in SD rats

**Figure 3:**
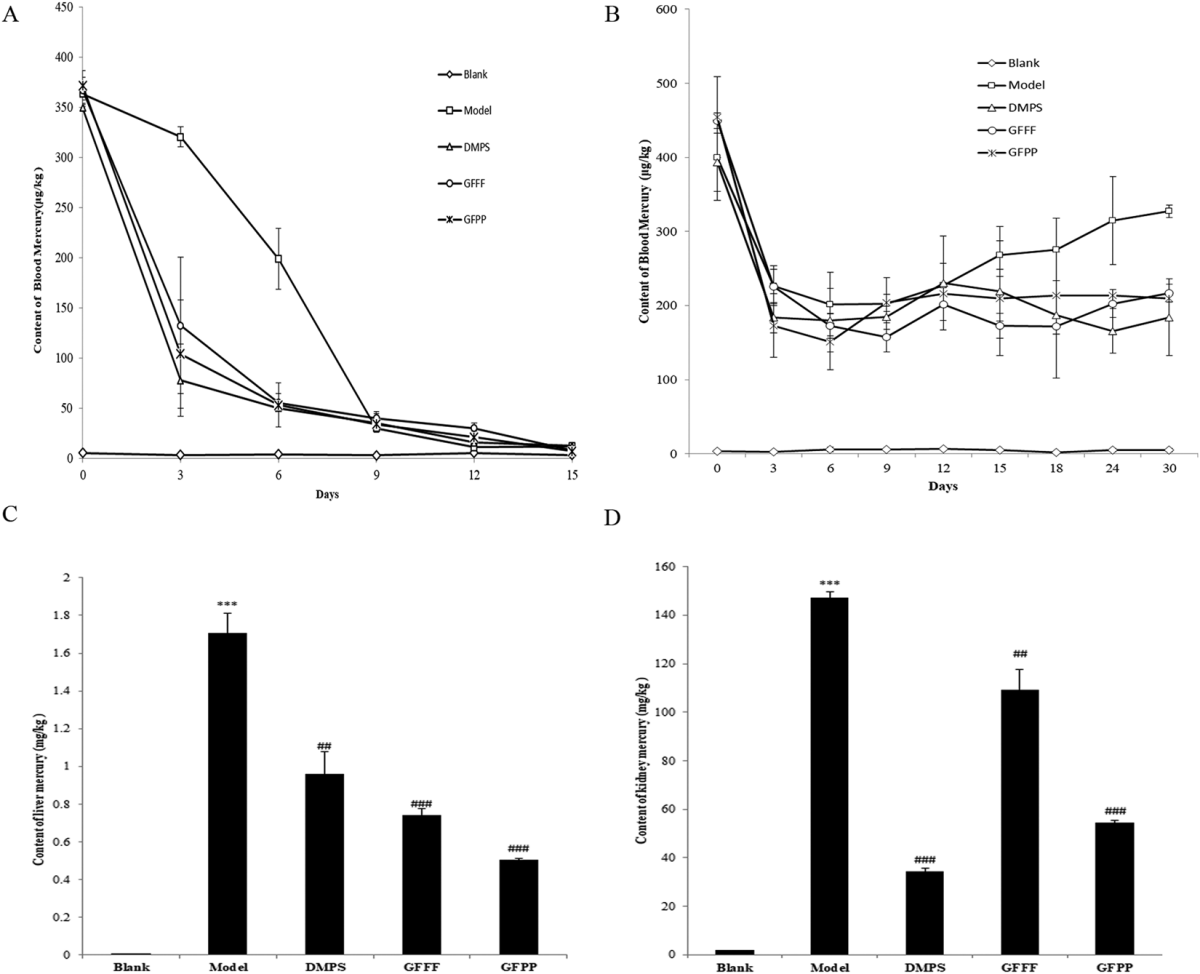
The clearance effect of GFPP and GFFF on (**A**) blood mercury level in acute HgCl_2_ poisoning rats (**B**) blood mercury level of chronic HgCl_2_ poisoning rats (**C**) The content of mercury in liver and in (D) kidneys in acute mercury poisoning rats receiving different treatments. Compared with blank group, ^***^p < 0.001, ^**^p < 0.01, ^*^p < 0.05; compared with model group, ^###^p < 0.001, ^##^p < 0.01, ^#^p < 0.05 (n = 6).

SD rats were acutely poisoned with HgCl_2_ at a concentration of 1100 μg/kg body weight for 3 days. The poisoned rats in the model group exhibited yellowish hair, a poor appetite, weight loss and low spirit and activity. The blood mercury levels were monitored for 15 days and are displayed in Fig. 3A. It is noteworthy that different concentrations of GFPP and GFFF were detected and better detoxication could be achieved at higher concentrations of GFPP and GFFF. For better presentation effects, rats treated with low concentrations of GFPP and GFFF were excluded from the figure. The blood mercury levels in all mercury-treated groups were significantly higher than that of the normal rats after 3 days of mercury administration. After oral administration of DMPS, GFPP or GFFF, the blood mercury level decreased faster than the model group. The blood mercury level in the model group was reduced to half on the 7^th^ day whereas groups treated with GFPP, GFFF and DMPS, the blood mercury level was reduced to less than half on the second day. The mercury removing effects of GFPP and GFFF were close to that of the drug DMP which served as a positive control. The blood mercury levels of all the groups included model group declined in exactly the same way as control group after 15 days which indicates that blood mercury could be eliminated through self-metabolism to alleviate the toxic action.

The rat liver and kidneys were also tested for accumulation of mercury (Fig. 3C,D). The results showed that mercury accumulated in the kidneys to a much higher extent than that in the liver, in keeping with reports in the literature^32,33^. Compared with the untreated rats, the mercury content in both the liver and kidneys of mercury-treated rats were significantly higher. Rats treated with the clinical drug DMPS exhibited obvious mercury clearance effect in the liver and kidneys, especially in the kidneys, in accordance with previous reports^34,35^. Both GFPP and GFFF reduced the accumulation in the liver and kidneys. GFPP exhibited a better curative effect than GFFF, even better than that of DMPS in the liver. The results disclose that *G. frondosa* could reduce the burden of mercury in the liver and kidneys.

### Facilitating effect on mercury excretion in chronic HgCl_2_ poisoning rats

From the previous experiment, the peak of blood mercury level appeared after three days of injection of HgCl_2_ at a dosage of 1100 μg/kg body weight. To establish a rat model of chronic poisoning, HgCl_2_ at a dosage of 600 μg/kg body weight was injected daily. Blood sample was taken every three days and the mercury concentration determined. The results of all the groups are displayed in Fig. 3B. Compared with the blank group, the blood mercury levels of all the other groups were significantly higher. The level of blood mercury were significantly higher in in all the mercury-treated than the blank group appeared after 3 days of mercury injection. But it reduced by more than half on the third day and remained stable thereafter. This indicated that mercury could be eliminated from the blood by metabolism. From the 12^th^ day onward, the blood mercury level in the model group presented an increasing trend, which indicated mercury could not be excreted by metabolism. Whereas in groups treated with DMPS/GFPP/GFFF, the blood mercury level was maintained within a relatively stable range. By the 30^th^ day, there was a significant difference (p < 0.05) in blood mercury level between the model group and other groups receiving HgCl_2_ injection. Moreover, the significant difference of GFPP- and GFFF- treated groups versus the model group was higher than that of DMPS-treated group versus the group, indicating that both GFPP and GFFF could act like DMPS, to facilitate removal of mercury from the blood.

## Discussion

As a heavy metal with diverse applications, mercury is ubiquitous in our surroundings. Several reports about the capacity of edible mushrooms in adsorption of heavy metal ions^36,37^ reminds that edible mushrooms may possess substances which could absorb mercury. Through conducting an *in vitro* assay on 36 normal cultivated mushrooms, we observed that the mercury absorbance of crude extracts from *Grifola frondosa* was higher and much more stable than those of other mushrooms, which motivated us to investigate the active component and the *in vivo* effect.

A crude fraction of *Grifola frondosa* fruiting bodies obtained through hot water extraction and alcohol precipitation had a mercury clearance rate of 56% *in vitro*, and this active component was thermostable. Ion exchange chromatography and gel filtration chromatography were applied to yield homogeneous components. The absorbance at 280 nm and 490 nm through the phenol sulfuric acid assay indicates this component may be a polysaccharide-peptide. The results of SDS-PAGE indicated a molecular mass of about 6 kDa for GFPP which was obviously smaller than the whole molecular mass of GAPP, indicating that the polysaccharide chain is the major part of GFPP. A ten-amino-acid sequence at the N-terminal of GFPP was determined. Seven high reliability peptide sequences were also obtained by ESI-MS/MS. The mechanism of binding between GFPP and mercury is not clear. Functional groups such as hydroxyl, carboxyl, carbonyl, sulfydryl, and amidogen have been reported to form mercury complexes^38–40^. Amino functional groups contribute to the binding of heavy metals whereas carboxyl or hydroxyl groups are involved in the formation of coordination bonds that confer stability to metal ion polymers^41,42^. There were also studies which revealed that microbial exopolysaccharides are highly capable of binding with metal ions due to the presence of numerous binding sites of negatively charged functional groups associated with proteins and polysaccharides^38,39^. We speculated this might be the reason of mercury adsorbance of GFPP since there is an abundance of these functional groups in the amino acid side chains and polysaccharides (mostly 50% higher in peptide, and even 80% higher in N-terminal sequence).

The mercury clearance ratio of purified GFPP rose as the concentration increased. At the same concentration, mercury clearance ratio rose after each purification step but not in proportion with the purification fold. Fractions from each purification step also exhibited mercury clearance ability even not as high as that of GFPP. These components may work synergistically in mercury absorption.

The *in vivo* mercury clearance effect of GFPP was testified by employing HgCl_2_ intoxication rat models. Two models were established in this study: the rat model of acute poisoning and the rat model of chronic poisoning, to simulate intoxication cases. The fruiting body powder was also tested for its curative effect. Blood mercury level could be decreased by metabolism several days after discontinuation of exogenous mercury in the acute poisoning model. However GFPP and GFFF intake could significantly reduce the time taken and help blood mercury quickly return to the normal level. The mercury clearance effect could also be observed in rats subjected to chronic poisoning in which blood mercury level remained stable whereas blood mercury level of the model group rose continuously. The liver and kidneys are the two main organs in which mercury accumulates^43^. The results show that GFPP and GFFF could act like DMPS in decreasing the content of mercury in the liver and kidneys. Since DMPS has adverse effects and is thus not desirable for long-term usage^44^, natural products may be a new source of antidotes, especially for individuals exposed to mercury. Besides, polysaccharides from edible fungi manifest hepatoprotective and antioxidant effects which could antagonize oxidative effects produced by mercury. GFPP purified in this study demonstrates mercury clearance ability both *in vivo* and *in vitro*, and similar preparations have been reported to possess antioxidant effect in previous studies^45,46^. In addition, the specific affinity of GFPP toward mercury enables it to facilitate the excretion of mercury without loss of other necessary elements. Thus GFPP has the potential to be developed into a mercury antidote for acute poisoning and a health care drug for elimination of heavy metals and an antioxidant for constant use.

In this study, *Grifola frondosa* fruiting bodies displayed the ability of mercury clearance in rats. According to the equivalent dose conversion between animals and humans in pharmacological tests, consumption of 7.44 g dried fruiting body or 74.4 g fresh fruiting bodies could be enough for promoting the excretion of mercury in an adult with a body weight of 60 kg.

## Methods

All experimental protocols were approved by the University Safety Office and Animal Experimentation Ethics Committee at The Chinese University of Hong Kong and China Agricultural University. All experimental procedures were carried out in accordance with the approved guidelines of the Animal Care and Use Committee of The Chinese University of Hong Kong and China Agricultural University.

### Materials

Dried fruiting bodies of *Grifola frondosa* that we cultivated were used for this investigation. The strain is preserved in the Biological Fermentation Laboratory at the Institute of Medicinal Plant Development, Medical Collage Chinese Academy of Medical Sciences & Peking Union. Sprague Dawley rats weighing 250–300 g were purchased from Beijing Huafukang Biosource Company (SCKK 2014-0004). There were no significant differences in body weight among the animals used for the studies.

### Extraction of polysaccharide-peptide from *G. frondosa* fruiting body

Dried fruiting bodies of *Grifola frondosa* were homogenized in deionized water (mass to volume ratio = 1:10) and incubated for 6 hours at 95 °C. After centrifugation at 9000 rpm for 10 min, the sediment was repeatedly incubated with deionized water (mass:volume = 1:1) at 95 °C for 3 hours for better extraction. The water extract of *G. frondosa* was obtained by combining the supernatants from two centrifugation runs and its volume was reduced by using vacuum-rotary evaporation. This water extraction was then alcohol precipitated by adding 4 times fold volume of alcohol and standing overnight. The crude *G. frondosa* polysaccharide peptide (GFPP) was enriched in the sediment after centrifugation of the mixture and then dried at 45 °C and stored for future use.

For isolation of GFPP, an anion exchange chromatography column (DEAE-cellulose) was pre-equilibrated thoroughly with deionized water. Crude GFPP powder was dissolved and centrifuged at 9000 rpm for 10 min. The supernatant was loaded on the column which was eluted with 0 mM, 50 mM, 150 mM and 1 M NaCl sequentially at a flow rate of 2 mL/min. The effluent fraction was collected in tubes (2 mL per tube) and polysaccharide concentration and protein concentration were determined by using phenol-sulfuric acid method (A490 nm)^47^ and UV spectrophotometry (A280 nm), respectively. Based on the elution profiles, four factions were collected separately. Fraction D4 was proved to have highest mercury-removing capacity. D4 was then loaded on a Superdex 75 column for gel filtration through an AKTA Purifier system.

### SDS-PAGE of GFPP and sequence analysis

Purified GFPP was dissolved in 10 μL ddH_2_O, and mixed with 2× tricine-SDS-PAGE loading buffer. The mixture was cooled immediately after boiling for 5 minutes. The sample was loaded on a Novex™ 16% Tricine Protein Gel and electrophoresed under 80 V for 1.5 hours according to the manufacturer’s instructions. The gel was then stained and destained according to the manufacturer’s instructions. The band was analyzed by Edman sequencing and ESI-MS/MS in the Center of Biomedical Analysis, Tsinghua University.

### Determination of *in vitro* mercury clearance ratio

For samples from the various purification steps, each of the fractions was dissolved in deionized water at a final concentration of 4 mg/mL. HgCl_2_ was diluted with HNO_3_ solution (10%) to yield a final concentration of 2 ppm. A mixture of equal volumes of a sample and HgCl_2_ solution was vibrated at 150 rpm for 2 hours at room temperature. Four volumes of alcohol were added to the reaction mixture to precipitate the polysaccharides and the supernatant was obtained by centrifugation at 9000 rpm for 10 min. The supernatant was diluted appropriately for determination of the concentration of Hg by Atomic Fluorescence Spectroscopy (AFS).

The clearance ratio was calculated as follows:

$${\rm{Clearance}}\,{\rm{ratio}}( \% )=\frac{{\rm{Concentration}}\,{\rm{in}}\,{\rm{control}}\,{\rm{group}}-{\rm{Concentration}}\,{\rm{in}}\,\mathrm{experiment}\,\,{\rm{group}}}{\,\mathrm{concentration}\,{\rm{in}}\,{\rm{control}}\,{\rm{group}}}\times 100$$

Samples from rats, such as blood, liver and kidney were subjected to microwave digestion before determination of mercury content. Blood sample (0.5 mL) or organ powder (0.5 g) was placed in a digestion tank and HNO_3_ (5 mL) and H_2_O_2_ (2 mL) were added. The digestion tank was located in a microwave digestion system and the program was started in accordance with the manufacturer’s instructions. Upon completion of the protocol, the sample in the tank was diluted into 50 mL and used for determination of mercury content using AFS.

### Assay of affinity for different metal ions

To evaluate the metal ion affinity of GFPP, 4 mg/mL of GFPP was mixed with an equal volume metal ions mixture of various metal ions including Mg^2+^, Al^3+^, Ca^2+^, Mn^2+^, Cu^2+^, Fe^2+^,Cr^2+^, Cd^2+^, Pb^2+^and Hg^2+^ ions, with a concentration of 2 ppm for each ion. The reaction process was as mentioned above. The supernatant was determined of the concentration of each metal ions by ICP-MS (Thermo Fisher).

### Preparation of reagents related to animal experiments

Pentobarbital sodium (3% solution) was used for anesthesia of rats. DMPS (5.26 mg/mL) was used as a positive control since it has a remarkable mercury removing capacity. GFPP was dissolved at a concentration of 12 mg/mL. *G. frondosa* fruiting body powder was mixed with basal feed powder and remade manually into *G. frondosa* fruiting body feed (GFFF) at a final concentration of 1% and 5%, respectively.

### Removal of mercury in SD rats that experienced acute mercury poisoning

The SD rat model of acute mercury poisoning was established by intraperitoneal injection of HgCl_2_ solution at the dosage of 1,100 μg/kg body weight daily for 3 days in succession. GFPP and GFFP was oral administered orally after 2 days of recovery. Forty-two SD rats were divided into seven groups with 6 rats per group. The seven groups received different treatments as shown in Table 3.

**Table 3:** The methods of grouping and acute administration of mercury in rats.

Group	Acute poison	Samples treated	Methods and dosages
Blank group	No	ddH_2_O	Intragastric administration (0.8 mL/100 g body weight)
Model group	Yes	ddH_2_O	Intragastric administration (0.8 mL/100 g body weight)
DMPS group	Yes	DMPS	Intragastric administration (0.8 mL/100 g body weight)
GFFF Low-dosage treated group	Yes	Artificial feed contains GFFF (1%)	Oral feeding (1.7 g/100 g body weight)
GFFF High-dosage treated group	Yes	Artificial feed contains GFFF (5%)	Oral feeding (1.7 g/100 g body weight)
GFPP Low-dosage treated group	Yes	GFPP solution 12 mg/mL	Intragastric administration (0.8 mL/100 g body weight)
GFPP High-dosage-treated group	Yes	GFPP solution 48 mg/mL	Intragastric administration (0.8 mL/100 g body weight)

The blood samples of the rats were collected from the eye socket vein six times, before administration and after administration for 3, 6, 9, 12 and 15 days. At least 0.5 mL blood from each rat was collected into a microextraction tube which contained heparin sodium and shaken repeatedly to avoid blood coagulation. All blood samples were preserved at −20 °C. Rats were anesthestized to death after the last blood collection. The liver and kidneys were collected, rinsed with saline, freeze-dried and pulverized into powder. Both blood samples and organ powders were nitrolyzed and determined for mercury concentration using Atomic Fluorescence Spectroscopy.

### Mercury-removing effect in SD rats that experienced chronic mercury poisoning

The SD rat model of chronic mercury poisoning was established on the basis of the acute mercury poisoning model. HgCl_2_ at the dosage of 1,100 μg/kg body weight was injected intraperitoneally daily for 3 days. After recovery for 2 days, HgCl_2_ (600 μg/kg body weight) was given by intraperitoneal injection. GFPP and GFFP were administered orally 2 to 3 hours later after the intraperitoneal injection of HgCl_2_ on each day. Blood samples was collected once every three days and the whole experimental period lasted for 30 days. The groups of rats were treated as summarized in Table 1 but groups given low concentration GFPP and GFFP were no longer included due to its better mercury removing rate.

### Statistical analysis

All test data were statistically analyzed by SPSS 20.0 software. ANOVA single factor analysis of variance was used. All results were presented as mean ± SD.
